# Cannibalism in *B* *uchananiella whitei* (Hemiptera: Anthocoridae) and its intraguild interactions with seven predatory mites (Acari: Phytoseiidae & Laelapidae)

**DOI:** 10.1002/ps.71042

**Published:** 2026-06-21

**Authors:** Yuzhi Gong, Keshi Zhang, Qiang Xuan, Zhi‐Qiang Zhang

**Affiliations:** ^1^ Manaaki Whenua ‐ Landcare Research Group Bioeconomy Science Institute Maiangi Taiao Auckland New Zealand; ^2^ School of Biological Sciences, University of Auckland Auckland New Zealand; ^3^ State Key Laboratory of Palaeobiology and Stratigraphy, Nanjing Institute of Geology and Palaeontology, Chinese Academy of Sciences Nanjing China; ^4^ University of Chinese Academy of Sciences Beijing China

**Keywords:** biological control, extraguild prey, predator mites, insect, Phytoseiidae, predator interaction

## Abstract

**BACKGROUND:**

Cannibalism and intraguild predation (IGP) are common interactions among predators that can influence the effectiveness of biological control agents. The minute pirate bug *Buchananiella whitei* is a recently commerciali sed biocontrol agent in New Zealand, but its intraspecific predation and interactions with potentially co‐occurring predatory mites remain poorly understood. This study examined cannibalism within *B. whitei*, as well as IGP between first‐instar *B. whitei* nymphs and adult females of seven predatory mite species (Phytoseiidae and Laelapidae), using observations in enclosed setups with emphasis on the role of extraguild prey availability (dried fruit mite *Carpoglyphus lactis*).

**RESULTS:**

Both cannibalism and IGP were observed, and their occurrence was strongly influenced by extraguild prey availability. No predation on *B. whitei* eggs was observed in either cannibalism or IGP. Cannibalism occurred only in adult‐nymph interactions, and its prevalence was greatly reduced by the presence of extraguild prey. IGP was more frequent in the absence of extraguild prey. First‐instar *B. whitei* nymphs preyed on most predatory mite species, but reciprocal predation was only observed with *Neoseiulus cucumeris* and *Stratiolaelaps scimitus*. Differences in body size among predator species partly contributed to the observed outcomes.

**CONCLUSION:**

These findings indicate that although cannibalism and IGP can occur in systems involving *B. whitei*, their ecological significance is probably limited when alternative prey are available. Predatory mites are therefore unlikely to substantially suppress *B. whitei* populations. A better understanding of these trophic interactions will improve the use of *B. whitei* and other natural enemies in biological control programs. © 2026 The Author(s). *Pest Management Science* published by John Wiley & Sons Ltd on behalf of Society of Chemical Industry.

## Graphical abstract

Extraguild prey reduces cannibalism and intraguild predation in *Buchananiella whitei*, while first‐instar nymphs dominate most predatory mites, with outcomes affected by body size and behavioral traits.

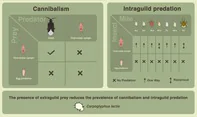

## 1 INTRODUCTION

Biological control, a cornerstone of sustainable agriculture, utilizes natural enemies—including parasitoids, predators, and microorganisms—to suppress pest populations.^1^, ^2^, ^3^ These control agents are widely used in greenhouse and field crops, including tomato, sweet pepper, strawberry, and ornamental plants.^4^, ^5^, ^6^, ^7^ Among predatory agents, predatory bugs are particularly effective against aphids, psyllids, thrips, and whiteflies.^4^, ^5^, ^6^, ^7^, ^8^, ^9^ In New Zealand, the minute pirate bug *Buchananiella whitei* (Reuter) (Hemiptera: Anthocoridae), an endemic species distributed across multiple regions,^10^, ^11^ has been commercialized for the control of a wide spectrum of arthropod pests.^12^ Its effectiveness is still under investigation, but its potential has been demonstrated against certain pest species (*e.g*., spider mites and thrips).^13^, ^14^ The biology and distribution of *B. whitei* are poorly known, but its eggs have been observed alongside leaf veins (Gong Y., pers. obs.).

Cannibalism—the predation of conspecifics—is a widespread phenomenon among predators and is often influenced by food limitation.^15^, ^16^, ^17^ Cannibalistic individuals gain nutrients while simultaneously reducing intraspecific competition for resources such as food, space, and mates. Cannibalism has been observed in other predatory bug species belonging to the family Anthocoridae (*e.g*., *Orius laevigatus* and *Orius strigicollis*),^18^, ^19^, ^20^ with potential implications for both population dynamics and biocontrol efficiency.

Intraguild predation (IGP)—the consumption of heterospecific predators sharing the same prey—is also widespread in the animal kingdom and represents a combination of competition and predation.^21^, ^22^, ^23^, ^24^, ^25^ IGP provides direct nutritional benefits while reducing competition for shared resources.^24^ Its likelihood increases under conditions of food scarcity, high predator densities, or frequent encounters among predators, and its strength is often influenced by factors such as relative body size, feeding mode, diet, life‐history traits, and habitat.^22^, ^23^, ^24^ In biological control systems, the release of multiple predator species or the presence of naturally occurring predators at release sites can expose control agents to IGP, potentially affecting pest suppression.^26^, ^27^, ^28^, ^29^, ^30^

Predatory mites (Acari: Mesostigmata) are widely found, and are key components of both natural and augmentative biological control.^31^ The family Phytoseiidae predominantly comprises free‐living, plant‐inhabiting predators, many of which are extensively employed against agricultural pests.^32^, ^33^ Notable examples include *Amblydromalus limonicus* (Garman & McGregor), *Neoseiulus californicus* (McGregor), and *Neoseiulus cucumeris* (Oudemans) which are used to manage a broad range of insect and mite pests in agricultural systems, such as scale insects, thrips, whiteflies, and spider mites.^32^, ^34^, ^35^, ^36^, ^37^, ^38^, ^39^, ^40^ A few soil‐dwelling predatory mites, particularly within the family Laelapidae, have been effective at controlling soil pests.^2^ Among generalist soil predators, *Stratiolaelaps scimitus* (Womersley) is notable for its efficacy against diverse pests, including fungus gnats, thrips, mold mites, and nematodes.^2^, ^41^, ^42^, ^43^, ^44^

Intraguild predation appears to be more prevalent among generalist predatory mites than among specialists.^45^ Thus, we examined IGP between *B. whitei* and seven predatory mite species that are either widely distributed in New Zealand or commonly used as biological control agents, and therefore may overlap with *B. whitei* in both natural and managed systems.^46^, ^47^, ^48^, ^49^ These predatory mites were selected to represent a range of habitats and plant microhabitats, including glabrous foliage (*e.g*., *Am. limonicus*), moderately pubescent foliage (*e.g*., *Amblyseius herbicolus* [Chant] and *Amblyseius lentiginosus* Denmark & Schicha), highly pubescent foliage (*e.g*., *Phytoseius leaki* Schicha), litter and soil habitats (*e.g*., *N. cucumeris* and *S. scimitus*), and habitats containing dense spider mite webbing (*e.g*., *N. californicus*).^32^, ^46^, ^50^, ^51^ Although the habitat preference of *B. whitei* remains unknown, its potential to suppress shared pests, such as spider mites and thrips, may provide opportunities for interaction with these predatory mites.^13^, ^14^

Since food availability is one of the main factors affecting the prevalence of both cannibalism and IGP,^24^, ^52^ we examined the influence of extraguild prey availability on cannibalism within *B. whitei* and on IGP between *B. whitei* and the examined predatory mites. The dried fruit mite *Carpoglyphus lactis* (Linnaeus) (Acari: Carpoglyphidae) is a common pest but is also widely used as factitious prey for rearing predatory mites and *B. whitei*.^40^, ^46^, ^50^, ^53^, ^54^, ^55^ Because all predatory species used in the present study are capable of feeding on *C. lactis*, this species provided a standardized alternative prey source for comparing intraguild interactions among predators. Additionally, *C. lactis* is commonly supplied as supplemental food to maintain predatory mite populations on plants.^56^

We expect both cannibalism and IGP between *B. whitei* and co‐occurring predatory mites are expected to occur. Thus, we hypothesized that (i) eggs of *B. whitei* might be vulnerable to predation by conspecifics, particularly adults, as well as by predatory mites, and (ii) the occurrence of cannibalism and IGP would be modulated by resource availability, with the provision of extraguild food reducing the frequency of these interactions. To test these hypotheses, we quantified egg predation of *B. whitei* by first‐instar *B. whitei* nymphs, adult conspecifics, and adult female predatory mites. Cannibalism assays were performed using either a conspecific first‐instar nymph or an adult *B. whitei*, whereas one gravid female predatory mite was used in IGP assays. To examine the influence of food availability, experiments were conducted in the absence and presence of *C. lactis* prey. This study provides implications for optimizing the use of *B. whitei* and other natural enemies in biological control programs.

## 2 MATERIALS AND METHODS

### 2.1 Study subjects and rearing conditions

**Figure 1:**
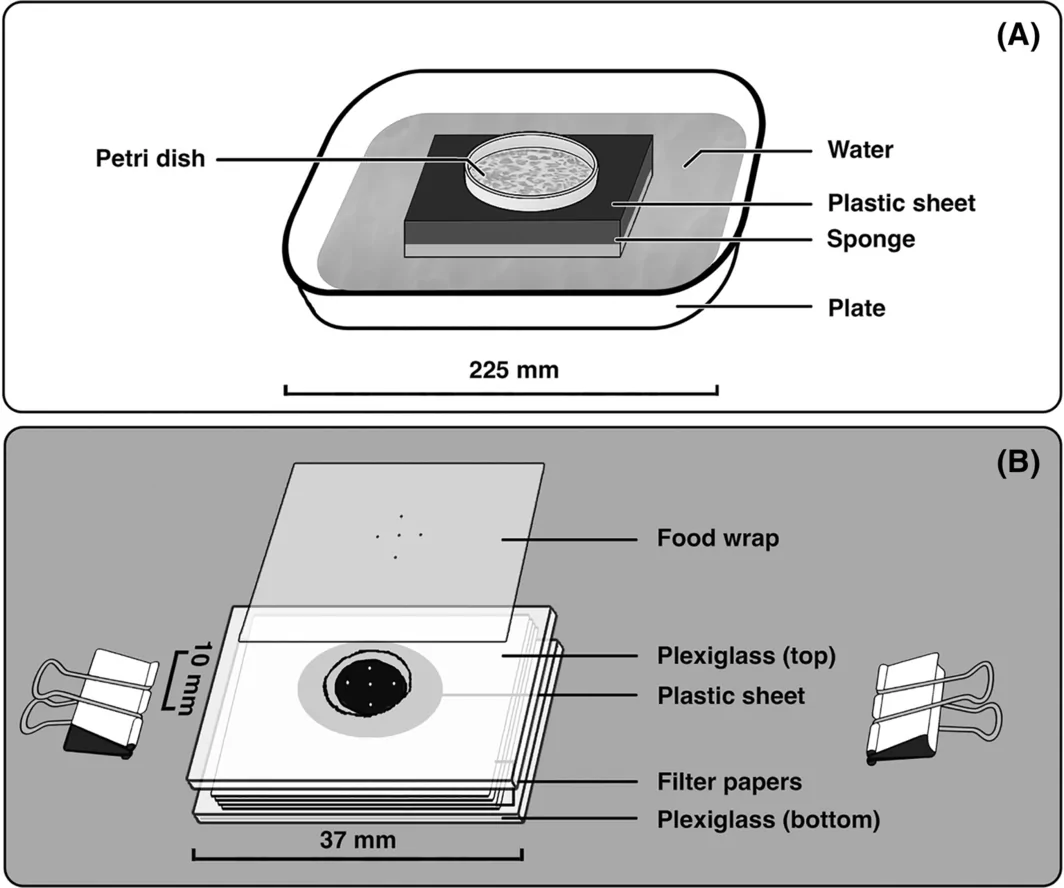
Experimental setup used in this study: (A) rearing units of mites; (B) experimental arenas.

*Buchananiella whitei* was obtained from Bioforce Limited (Karaka, Auckland, New Zealand) (see Gong *et al*.^12^ for details of the rearing method). All mites were collected or obtained in New Zealand and maintained under laboratory conditions (Table 1).^46^, ^49^ Rearing units comprised plastic containers filled with water, with a black plastic sheet placed on a sponge (Fig. 1A).^40^ All predatory mite species were reared on *C. lactis* with a bran‐sugar‐yeast mixture. Cultures and experimental units were maintained in plexiglass cabinets at 24 °C ± 1 °C, 80% ± 5% RH, and a 16:8 h light–dark cycle.

**Table 1:** Origins of the mite species used in this study

Role	Family	Species	Origin	Culture since
Predator	Phytoseiidae	*Amblyseius herbicolus*	Tauranga^a^	2024
		*Amblyseius lentiginosus*	Pleasant Point^b^	2023
		*Amblydromalus limonicus*	Hamilton^c^	2024
		*Neoseiulus californicus*	Auckland^d^	2025
		*Neoseiulus cucumeris*	Commercial supply^e^	2022
		*Phytoseius leaki*	Auckland^f^	2024
	Laelapidae	*Stratiolaelaps scimitus*	Commercial supply^e^	2025
Prey	Carpoglyphidae	*Carpoglyphus lactis*	Commercial supply^e^	2021

^a^ Found on avocado (*Persea americana* Mill.) leaves.

^b^ Found on the endemic New Zealand shrub *Teucrium parvifolium* (Hook.f.) Kattari & Salmaki.

^c^ Found on avocado (*Persea americana* Mill.) leaves.

^d^ Found on black nightshade (*Solanum nigrum* L.) leaves at Manaaki Whenua – Landcare Research, St Johns, Auckland.

^e^ Obtained in bulk from Bioforce Limited (Karaka, Auckland, New Zealand).

^f^ Found on woolly nightshade (*Solanum mauritianum* Scop.) leaves at Manaaki Whenua – Landcare Research, St Johns, Auckland.

The enclosed arenas used for experimental observations were constructed from two clear plexiglass slides (37 × 24 × 2 mm) (Fig. 1B).^57^ The top slide contained a centrally located, cone‐shaped hole (10 mm diameter at the top, narrowing to 7 mm at the bottom), forming a chamber of 115 mm^3^. This chamber was sealed with transparent food wrap for visibility, and a black plastic disc (20 mm diameter) was placed beneath to provide contrast for mite observation. Small perforations were made in both wrap and disc with a fine insect pin (size 000) to permit air and moisture exchange. Hydration was provided by a stack of filter papers beneath the disc. The bottom slide held the moisture reservoir, and the unit was secured with two foldback metal clips.

### 2.2 Bioassays

Predation experiments were conducted in the enclosed arenas, and filter papers beneath each arena were moistened with filtered water to sustain the survival of mites. Arenas were examined every 30 min for the first 3 h and again after 24 h to record predation outcome. All experiments were conducted under the same laboratory conditions as the main cultures.

#### 2.2.1 Egg predation

Egg cannibalism was assessed by placing either one newly hatched first‐instar nymph or one adult *B. whitei* in an arena with two conspecific eggs (<24 h old) (12 replicates). Egg predation by IGP predators was tested by offering two newly laid (<24 h old) eggs of *B. whitei* to a gravid female of each predatory mite species in the enclosed arena (12 replicates).

#### 2.2.2 Cannibalism

Cannibalism was examined by pairing one newly hatched first‐instar *B. whitei* with either a conspecific first‐instar nymph or an adult in an experimental arena. To test the influence of food availability, arenas were either left without food or supplied with prey consisting of 10 adult females of *C. lactis*. No oviposition of *C. lactis* was observed during the experimental period. For nymph‐nymph interactions, treatments were replicated 15 times with food and 20 times without food, whereas adult–nymph interactions were replicated 15 times under both food conditions. The occurrence of cannibalism and the surviving individuals were recorded.

#### 2.2.3 Intraguild predation (IGP)

IGP was evaluated by introducing one newly hatched first‐instar *B. whitei* nymph together with one gravid female predatory mite into the arena. To examine the effect of food availability on IGP, arenas were either left without food or supplied with extraguild prey consisting of 10 adult females of *C. lactis*. No oviposition of *C. lactis* was observed during the experimental period. Each treatment was replicated 8–12 times (Table 3). The occurrence of IGP and the prevailing species were recorded.

#### 2.2.4 Size comparison among predatory mites

Twenty adult females of each mite species were slide‐mounted for size measurements.^58^ The dorsal shield length was measured using a phase‐contrast microscope (Eclipse 90i, Nikon Corporation, Japan) with NIS‐Elements (version 5.10), which was used as an indicator of their body size. The dorsal shield is sclerotized in mites of Mesostigmata and a frequently used indicator of their body size.^59^, ^60^, ^61^

### 2.3 Statistical analysis

All statistical analyses were performed using R in RStudio (version 2023.12.1).^62^ Data visualization was carried out using the *ggplot2* package (version 3.4.3).^63^ For cannibalism, the occurrence of egg predation (yes/no) was analyzed using generalized linear models (GLMs) with binomial error distribution to assess the effect of life stage. The effects of life stage and prey availability on nymph cannibalism were analyzed using GLMs with binomial error distribution, including their interaction. For IGP, the occurrence of predation between *B. whitei* nymphs and predatory mites was analyzed using GLMs with a binomial error distribution. Model dispersions were examined to assess model fit. When IGP occurred, the outcome of predation (*i.e*., which species prevailed) was analyzed using binomial tests, assuming equal success probabilities for both species. The effect of prey availability on predation outcome was further examined using GLMs with binomial error distribution. For body size comparisons among predatory mite species, dorsal shield length was analyzed using analysis of variance (ANOVA), followed by Tukey's honestly significant difference test for pairwise comparisons. Statistical significance was set at *α* = 0.05.

## 3 RESULTS

### 3.1 Egg predation

Egg cannibalism by first‐instar nymph or adult *B. whitei* was never observed. Egg predation by intraguild predators (all seven species of predatory mites) was also not observed.

### 3.2 Cannibalism

Cannibalism differed significantly between life‐stage combinations (GLM: Wald *χ* ^2^ = 45.14, df = 1, *P* < 0.001). Specifically, cannibalism was never observed in nymph‐nymph interactions but occurred in adult–nymph interactions (Table 2). Extraguild prey presence significantly reduced cannibalism prevalence between adults and nymphs (Wald *χ* ^2^ = 8.58, df = 1, *P* = 0.003).

**Table 2:** Cannibalism between first‐instar nymphs and adults of *Buchananiella whitei* after 24 h. The adults of *B. whitei* were not sexed

Predator	Prey	Extraguild prey	*N*	Occurrence (%)
First‐instar nymph	First‐instar nymph	Yes	20	0%
		No	20	0%
Adult	First‐instar nymph	Yes	20	35%
		No	20	70%

### 3.3 Intraguild predation (IGP)

The occurrence of IGP was significantly affected by extraguild prey availability (GLMs: Wald *χ* ^2^ = 35.74, df = 1, *P* < 0.001), but not by mite species (Wald *χ* ^2^ = 6.58, df = 6, *P* = 0.362) or their interaction (Wald *χ* ^2^ = 6.14, df = 6, *P* = 0.408). Specifically, the presence of extraguild prey significantly reduced IGP prevalence (Table 3). When IGP occurred, *B. whitei* nymphs prevailed over *A. herbicolus*, *A. lentiginosus*, *A. limonicus*, *N. californicus*, *N. cucumeris*, and *P. leaki* (Table 3). However, females of *S. scimitus* were more prevailed than *B. whitei* nymphs (*P* < 0.001) (Table 3).

**Table 3:** Intraguild predation (IGP) outcomes between predatory phytoseiid mites and first‐instar nymphs of *Buchananiella whitei* after 24 h. One gravid adult female (<1 week old) of predatory mite and one first‐instar *B. whitei* nymph were placed in an enclosed cell in the presence (Yes) or absence (No) of five adult female dried fruit mites (*Carpoglyphus lactis*, extraguild prey)

Phytoseiid predator	Extraguild prey	*N*	Occurrence (%)	Survival rate (%)	
				Phytoseiid predator	1st instar nymph
*Amblyseius herbicolus*	Yes	10	20	0	100
	No	9	77.8	0	100
*Amblyseius lentiginosus*	Yes	9	33.3	0	100
	No	9	100	0	100
*Amblydromalus limonicus*	Yes	11	63.3	0	100
	No	8	100	0	100
*Neoseiulus californicus*	Yes	10	50	0	100
	No	10	80		100
*Neoseiulus cucumeris*	Yes	12	50	0	100
	No	12	100	33.3	66.7
*Phytoseius leaki*	Yes	8	37.5	0	100
	No	8	100	0	100
*Stratiolaelaps scimitus*	Yes	9	66.7	83.3	16.7
	No	10	90	100	0

### 3.4 Size comparison among predatory mites

**Figure 2:**
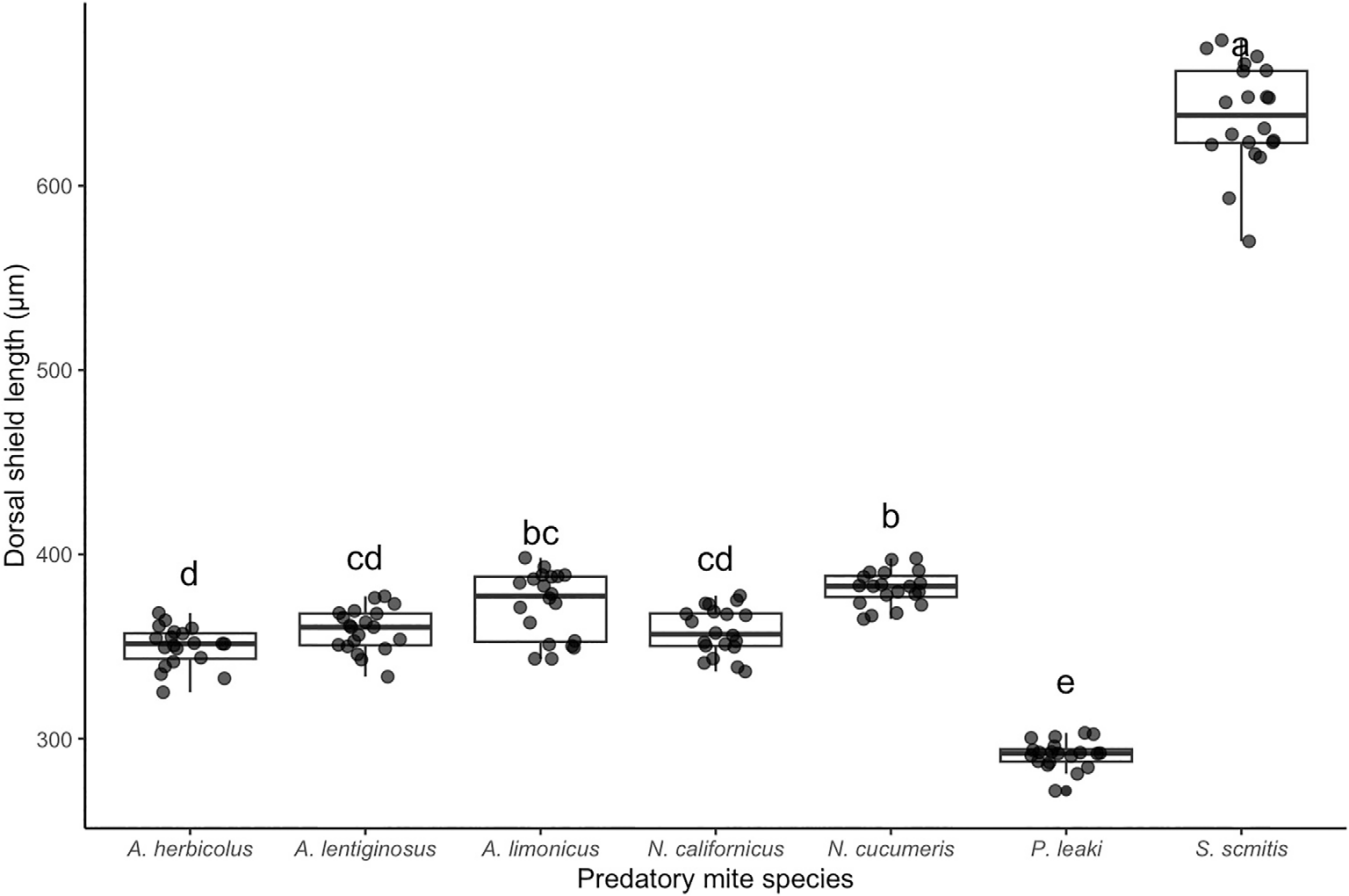
Body size (dorsal shield length) of predatory mite species: *Amblyseius herbicolus*, *Amblyseius lentiginosus*, *Amblydromalus limonicus*, *Neoseiulus californicus*, *Neoseiulus cucumeris*, *Phytoseius leaki*, and *Stratiolaelaps scimitus*. Boxes represent the interquartile range, horizontal lines indicate medians, and points show individual measurements. Letters above the boxes denote significant differences from pairwise comparisons (Tukey's test: *P* < 0.05).

Body size (dorsal shield length) differed significantly among predatory mite species (ANOVA: *F*₆,₁₃₃ = 1041, *P* < 0.001); *S. scimitus* was the largest species and *P. leaki* was the smallest (Fig. 2). *Neoseiulus cucumeris* was larger than all of the other species except *A. limonicus*.

## 4 DISCUSSION

Our results supported the hypotheses that cannibalism between *B. whitei* adult and first instar nymphs and IGP between *B. whitei* and predatory mites could occur, and that extraguild prey availability could significantly reduce their occurrence. The outcomes of predatory interactions were partly determined by relative body size. However, no individuals examined preyed on the eggs of *B. whitei*, suggesting that the egg capsule (chorion) of *B. whitei* may resist predation by conspecifics and predatory mites.^64^

Cannibalism occurred within *B. whitei*, but only between adults and first‐instar nymphs, whereas no cannibalism occurred between two first‐instar nymphs. Other studies have shown that cannibalism is often size‐dependent, with larger conspecifics preying on smaller ones.^24^, ^65^, ^66^ Nevertheless, the low prevalence of cannibalism observed in this study indicates low levels of aggression among conspecifics of *B. whitei*.

**Figure 3:**
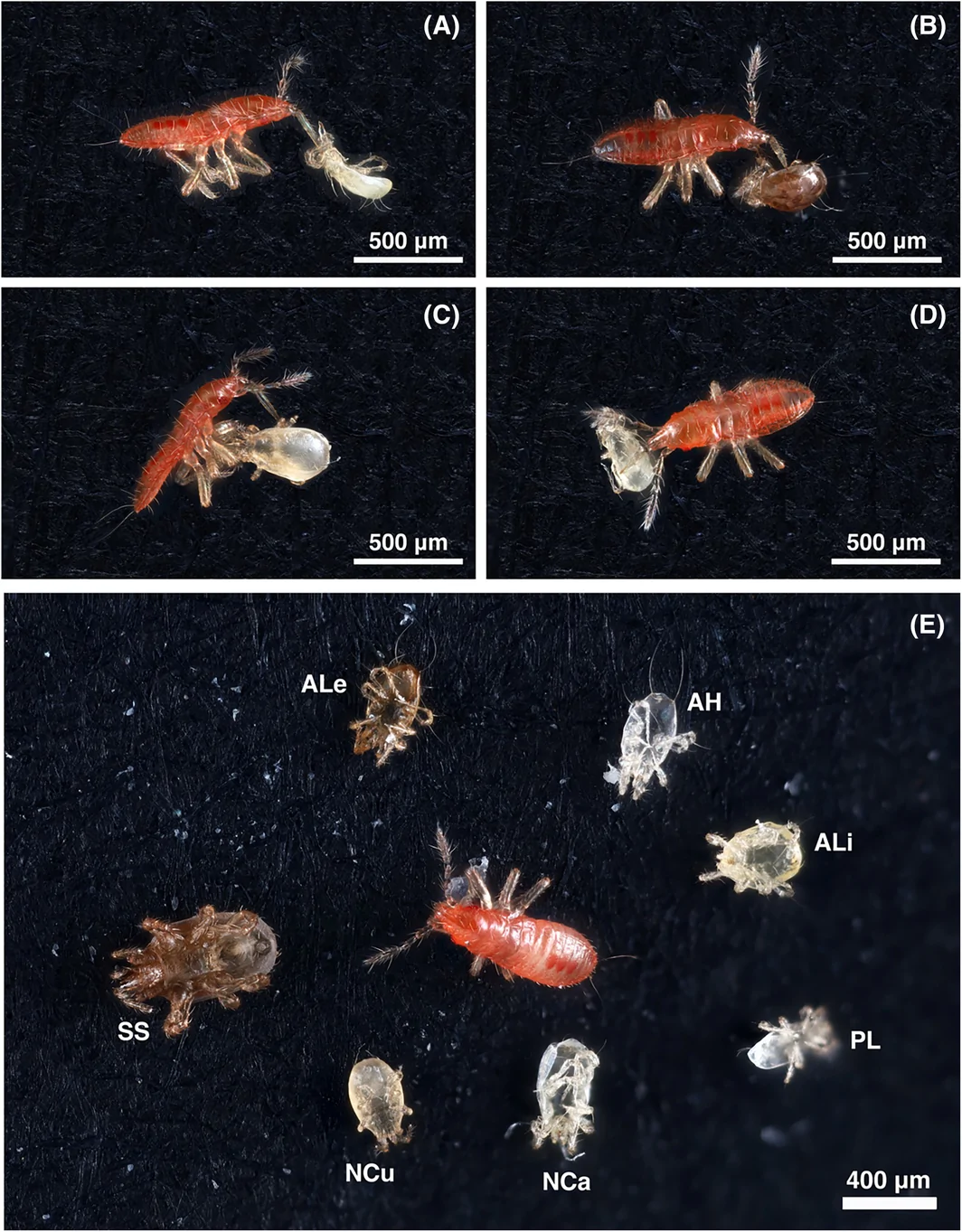
Intraguild predation by first‐instar nymphs of *Buchananiella whitei* on predatory mites. (A–D) Representative examples of predation events. (E) Size comparison between a first‐instar *B. whitei* nymph and different predatory mite species after predation. Abbreviations: ALe, *Amblyseius lentiginosus*; AH, *Amblyseius herbicolus*; ALi, *Amblydromalus limonicus*; PL, *Phytoseius leaki*; NCa, *Neoseiulus californicus*; NCu, *Neoseiulus cucumeris*; SS, *Stratiolaelaps scimitus*.

**Figure 4:**
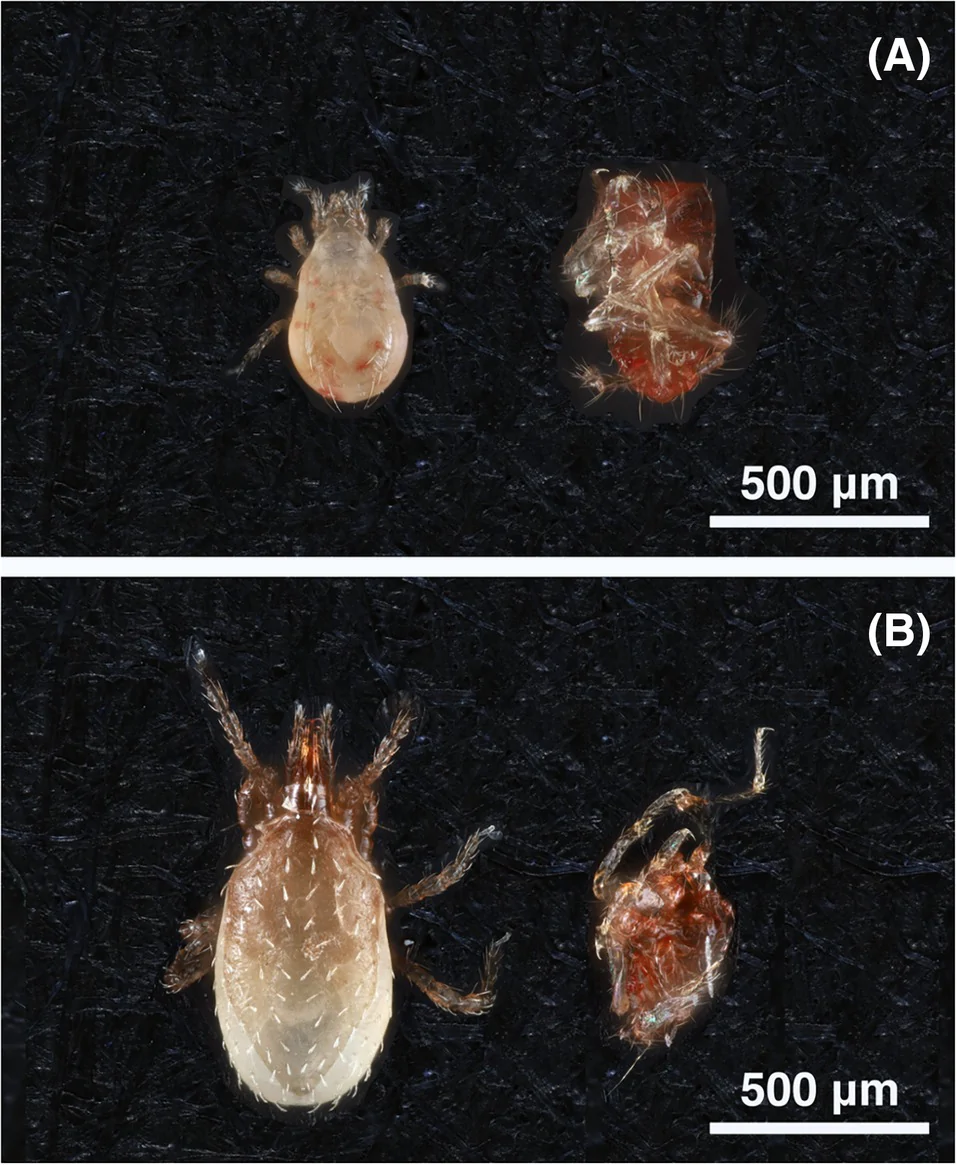
Size comparison between predatory mites and first‐instar nymphs of *Buchananiella whitei*. (A) *Neoseiulus cucumeris*; (B) *Stratiolaelaps scimitus*. In each panel, intact mites (left) are shown alongside consumed first‐instar *B. whitei* nymphs (right).

IGP was observed between first‐instar *B. whitei* nymphs and predatory mites, indicating that these species can function both as competitors and as predators within the same trophic system (Fig. 3). The outcome of IGP is probably explained by differences in body size, with larger predators typically dominating smaller ones.^24^, ^67^, ^68^ First‐instar *B. whitei* nymphs were larger than adult females of all examined predatory mites except *S. scimitus* (Fig. 3; Fig. 4). Although body size is an important determinant of IGP outcomes, it does not fully account for the observed patterns. Specifically, *N. cucumeris*, despite being smaller than *S. scimitus* and not larger than *A. limonicus*, was still able to engage in and occasionally succeed in IGP against *B. whitei* nymphs, suggesting that behavioral traits such as aggressiveness, attack efficiency, and escape ability may also play a critical role in determining IGP outcomes.^66^, ^69^, ^70^

We found that extraguild prey availability significantly reduced both cannibalism and IGP. This pattern is consistent with ecological theory and empirical studies on other arthropod species.^22^, ^65^, ^66^, ^71^, ^72^, ^73^, ^74^ For example, in predatory bugs *Orius insidiosus* and *O. laevigatus*, cannibalism and IGP prevalence declined with increasing food availability.^18^, ^19^ In addition to prey availability, previous studies on *N. californicus* and *N. cucumeris* have suggested that the type and quality of extraguild prey may also influence predator interactions, as prey suitability and nutritional quality can indirectly modify predator behavior and the occurrence of intraguild predation.^22^ Together, these findings indicate that although cannibalism and IGP can occur in *B. whitei*, they are less likely to have significant impacts under natural conditions. However, additional field‐based studies are required to confirm this.

**Figure 5:**
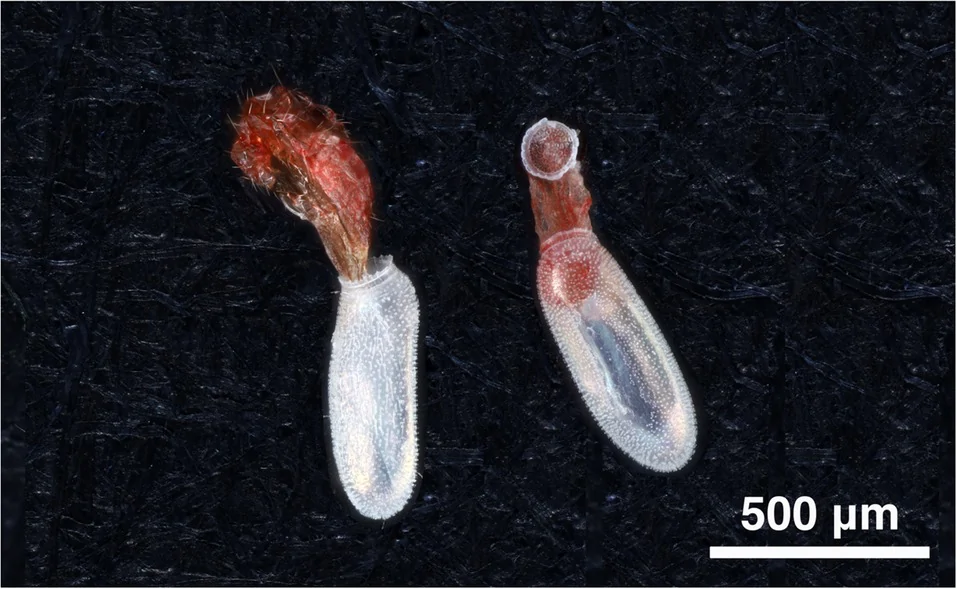
Observations of newly emerging first‐instar *Buchananiella whitei* nymphs consumed during hatching.

In this study, although no egg predation was observed, *B. whitei* may be vulnerable to predation during a very brief developmental window at hatching (c. 10 min) (Gong Y., pers. obs.). We occasionally observed predatory mites attacking newly emerged first‐instar nymphs during hatching, when individuals were relatively immobile and vulnerable (Fig. 5). Once fully emerged and active, first‐instar *B. whitei* nymphs were generally able to resist or avoid attacks from most predatory mites. This transient period of vulnerability may influence intraguild predation dynamics, oviposition site selection, and potentially the evolution of parental care behaviors, and warrants further investigation.

A limitation of this study is that all interactions were assessed under laboratory conditions in small, enclosed arenas, which probably increased encounter rates and may have overestimated the frequency of intraguild predation and cannibalism relative to field or greenhouse conditions. Consequently, the present results should be interpreted as evidence of interaction potential rather than direct predictions of post‐release outcomes.

Overall, the results suggest that predatory mites are unlikely to substantially suppress *B. whitei*, because egg predation was not observed, first‐instar *B. whitei* nymphs dominated most interactions with predatory mites, and both intraguild predation and cannibalism declined markedly in the presence of alternative prey. These findings have important implications for biological control. The strong reduction in antagonistic interactions under conditions of prey availability suggests that providing sufficient alternative prey could mitigate negative interactions among these natural enemies and improve the efficiency of target pest suppression.

## CONFLICT OF INTEREST DECLARATION

All authors declare no conflict of interest.

## Data Availability

The data that support the findings of this study are available from the corresponding author upon reasonable request.
